# BatMeth: improved mapper for bisulfite sequencing reads on DNA methylation

**DOI:** 10.1186/gb-2012-13-10-r82

**Published:** 2012-10-03

**Authors:** Jing-Quan Lim, Chandana Tennakoon, Guoliang Li, Eleanor Wong, Yijun Ruan, Chia-Lin Wei, Wing-Kin Sung

**Affiliations:** 1 Department of Computer Science, National University of Singapore, Singapore 117417; 2 NUS Graduate School for Integrative Sciences and Engineering, (CeLS), #05-01, 28 Medical Drive, Singapore 117456; 3 Genome Institute of Singapore, 60 Biopolis Street, #02-01 Genome, Singapore 138672; 4 Department of Biological Sciences, National University of Singapore, Singapore 117543; 5 Joint Genome Institute, Walnut Creek, CA 94598, USA

## Abstract

DNA methylation plays a crucial role in higher organisms. Coupling bisulfite treatment with next generation sequencing enables the interrogation of 5-methylcytosine sites in the genome. However, bisulfite conversion introduces mismatches between the reads and the reference genome, which makes mapping of Illumina and SOLiD reads slow and inaccurate. BatMeth is an algorithm that integrates novel *Mismatch Counting, List Filtering, Mismatch Stage Filtering *and *Fast Mapping onto Two Indexes *components to improve unique mapping rate, speed and precision. Experimental results show that BatMeth is faster and more accurate than existing tools. BatMeth is freely available at http://code.google.com/p/batmeth/.

## Background

DNA methylation modifies the nucleotide cytosine by the addition of methyl groups to its C5 carbon residue by DNA methyltransferases [1]. This modification can be inherited through cell division and it plays an important role in many biological processes, such as heterochromatin and transcriptional silencing [2,3], imprinting genes [4], inactivating the × chromosome [5] and silencing of repetitive DNA components in healthy and diseased (including cancerous) cells [6,7]. Methylation analysis can also be used to diagnose pre-natal Down's syndrome [8]. Thus, the genome-wide methylation profiles of different tissues are important to understand the complex nature and effects of DNA methylation.

In the past decade, quantum leaps have been made in the development of sequencing technologies by vendors such as Illumina-Solexa and Applied BioSystems (AB)-SOLiD. These can generate millions of short reads at a lower cost compared to traditional Sanger methods [9-13]. Bisulfite (BS) treatment converts unmethylated cytosines (Cs) to uracils (which are then amplified by PCR as thymine (T)) without affecting the other nucleotide bases and methylated cytosines [14]. Next-generation sequencing coupled with bisulfite treatment enables us to produce a methylome of a genome at single base resolution and low cost.

One important step in calling methylation of a genome is to map bisulfite reads. Mapping of bisulfite reads is different from that of ChIP-Seq and RNA-Seq data since the non-methylated Cs are converted to Ts by bisulfite treatment and subsequent PCR. The bisulfite reads are difficult to map to the reference genome due to the high number of mismatches between the converted Ts and the original Cs. For mapping Illumina bisulfite reads, the pioneering published methods are BSMAP [15] and RMAP [16]. BSMAP aligns a bisulfite read to the reference genome by first enumerating all C-to-T combinations within a user-defined length k seed of the reads; then, through hashing, BSMAP aligns the seeds onto the genome and putative alignments are extended and validated with the original reads. After this step, BSMAP can output an unambiguous hit for each read, if available. BRAT [17] uses a similar strategy as BSMAP. It converts the reference genome into a TA reference and a CG reference (each converted reference uses one bit per base). Using a 36-mer hash table, BRAT aligns the first 36 bases of every read and its 1-neighbors on the two converted references to identify possible alignments. RMAP uses layered seeds as a bit-mask to select a subset of the bases in the reads and constructs a hash table to index all the reads. However, these seed-hash-based approaches are slow.

Subsequently, several methods were proposed to map bisulfite reads onto the converted genomes. MethylCoder [18] surfaced as a bisulfite read mapper that uses GSNAP [19] to do a primary mapping of *in silico *converted reads (that is, all Cs in the reads are converted to Ts) onto a converted reference genome (that is, all Cs in the genome are converted to Ts). Those reads that fail to map onto the converted genome will be remapped again in their original forms onto the original reference. BS-Seeker [20] and Bismark [21] use a similar conversion strategy as BSMAP except that they align the reads with Bowtie [22] and unique hits are found by a seed-then-extend methodology. (Note that every tool has its own uniqueness criterion. A tool will denote a read to have a unique hit if it finds exactly one occurrence of the read in the reference genome.) Both methods trade accuracy for efficiency.

**Figure 1:**
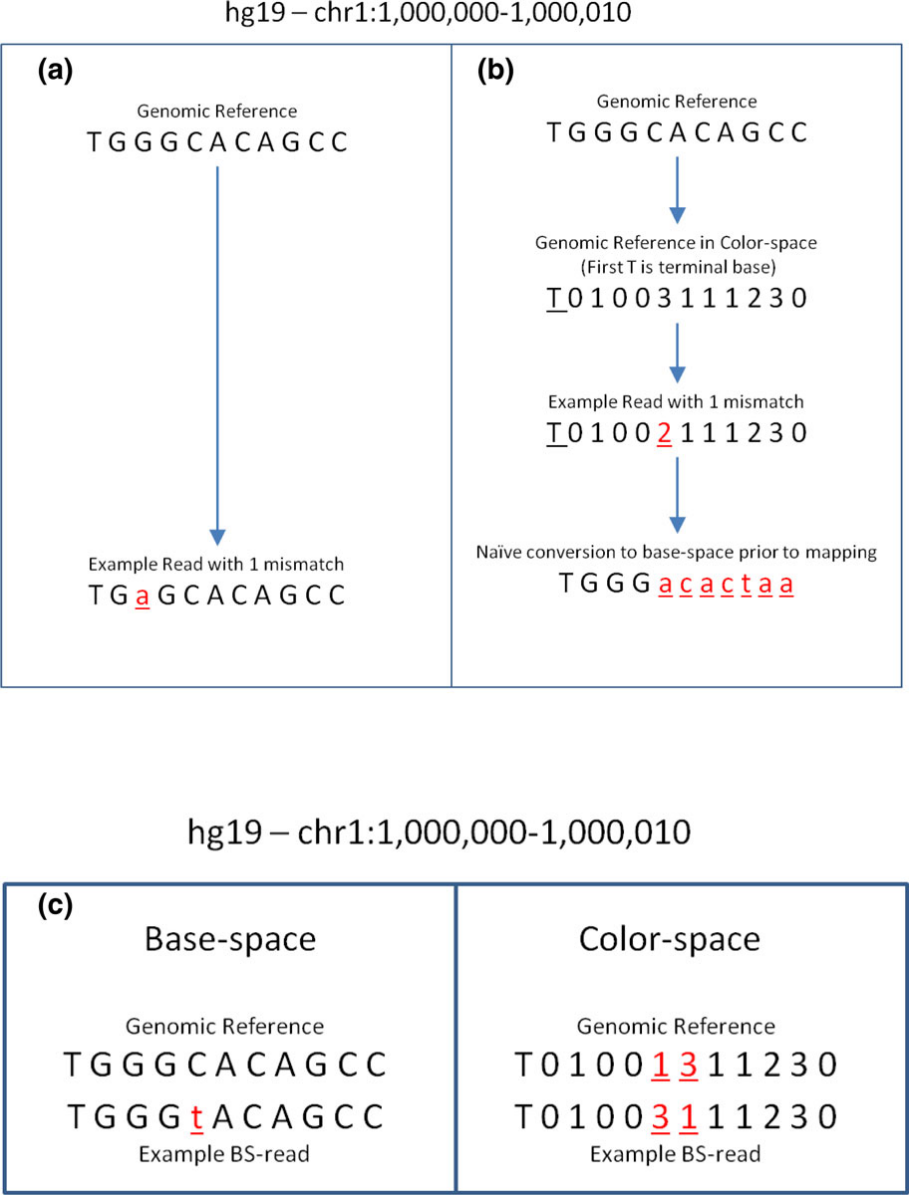
**Interpreting mismatches between reads in base- and color-space**. **(a,b) **Base call error simulation in Illumina and SOLiD reads reflecting one mismatch with respect to the reference from which they are simulated in their respective base- and color-space. (b) A naïve conversion of color read to base space, for the purpose of mapping against the base space reference, is not recommended as a single color base error will introduce cascading mismatches in base space. **(c) **A bisulfite conversion in base space will introduce two adjacent mismatches in its equivalent representation in color space.

AB-SOLiD color reads are different from Illumina reads since they encode every pair of bases with four different colors. (For more details on this sequencing technology and how it differs from sequencing by synthesis, see [23-26].) Unlike bisulfite mapping of Illumina reads onto converted genomes, mapping bisulfite color reads onto converted genomes produces many mismatches when the regions are highly methylated [27]. This also causes a dramatic decrease in the unique mapping rate and unbiased measurements of hypomethylation sites. In addition, a single color error in a read will lead to incorrect conversions throughout the rest of the read (Figure 1a,b). Although *in silico *conversion of Cs to Ts guarantees unbiased alignments in base space, this is not preferred for color reads.

SOCS-B [28] and B-SOLANA [29] were developed to map bisulfite color reads. SOCS-B splits a color read into four parts and tries to get hits for any combination of two parts via an iterative Rabin-Karp approach [30]. SOCS-B uses a dynamic programming approach to convert an aligned read to the aligned portion of the reference genome. The conversion starts with all possible four nucleotides as the pseudo-terminal base (rather than just the terminal base from the read). Subsequently, the sub-strings of the four translations are used to generate partial hashing seeds that are then mapped onto the hashed reference genome. However, the running time of SOCS-B is long and the unique mapping rate is too low to be practical. B-SOLANA improves speed and unique mapping rate by aligning against both fully converted and non-CpG converted references simultaneously with Bowtie. The final hits are determined by checking their number of mismatches.

A recent review article [27] reported that Bismark and BS-Seeker are the most recent published methods for mapping bisulfite base reads whereas B-SOLANA is the most recent published method for mapping bisulfite color reads. This review also highlighted the main challenges to develop methods that can map reads unbiasedly and to improve unique mapping rates for mapping color reads.

BatMeth (Basic Alignment Tool for Methylation) was developed by us to address the issues of efficiency and accuracy on mapping bisulfite reads from Illumina and bisulfite color reads from SOLiD. Unlike existing algorithms, BatMeth does not map the bisulfite reads in the initial stage. Instead, BatMeth counts the number of hits of the bisulfite reads to remove spurious orientations of a read. This idea has significantly sped up the mapping process and has also reduced the number of false positives. When dealing with color reads, BatMeth reduced bias on hypomethylation measurements with high initial mismatch scanning. BatMeth also employed a dynamic programming conversion step for the color reads to account for bisulfite mismatch accurately and an incremental processing step to produce higher unique mapping rates and speed (refer to the Materials and methods section for details).

We have compared the performance of BatMeth with recent stable versions of BSMAP (2.4.2), BS-Seeker, Bismark (0.5.4), SOCS-B (2.1.1) and B-SOLANA (1.0) using both simulated and real data sets (BS-Seeker, Bismark and B-SOLANA used Bowtie 0.12.7 in our experiments). With simulated Illumina and SOLiD reads, BatMeth (default mode) recovered the highest number of hits, has the lowest noise rate and is the fastest among the compared programs. BatMeth is also able to produce better unbiased results than the other programs by comparing the detected methylation levels in different genomic contexts over simulated data sets (Illumina and SOLiD reads) of different methylation levels. With a paired-end library, we show the specificity of our Illumina results by counting the pairs of concordant paired reads that fall within the expected insert size of the library. With a directional library, we indicate the specificity of our results with direction-specific information. In summary, BatMeth is an improved bisulfite mapper in terms of speed, recovery rate and accuracy, and, in particular, has addressed the main challenges of mapping color reads identified in [27].

## Results

### Evaluated programs and performance measures

In order to evaluate the performance of our pipeline, we have tested the following programs: BSMAP, BS-Seeker, and Bismark for base-space mapping; and SOCS-B and B-SOLANA for color-space mapping. BS-Seeker and Bismark only output unique hits for each read. BSMAP, SOCS-B and B-SOLANA will output at most one hit per read, with a flag to indicate if a hit is unique. Some reads can map to multiple genomic locations and since a read can only come from one origin, retaining such non-unique mappings will affect the accuracy of downstream analysis such as unbiased methylation site calls. To avoid the problem of wrong methylation calls, all six programs were thus compared with their unique mapping rates.

All our experiments were run on a server equipped with an Intel Xeon E7450 @ 2.40GHz and 128 GB of RAM. We allowed the same mismatch number and CPU threads on all the compared programs in our experiments. Other parameters were kept at default (see Section 1 of Additional file 1 for the choice of parameters used).

We have not included RMAP in our comparisons as it only performs biased mapping in a non-CpG context. MethylCoder was also not included because a newer variant of it, namely B-SOLANA, has been released (MethylCoder's release notes mention that it is now deprecated due to the release of B-SOLANA). BRAT was considered impractical as it only considers one base error in the first 36 bp of a read and therefore was not included in our experiments.

Below, we define 'recovery' to be the portion of the unique hits recovered by the programs. We also define 'accuracy' to be the portion of the recovered hits that are correct. All recorded timings are wall clock times. A 'hit' is a genomic location to which a read is aligned. Lastly, due to sequencing errors and bisulfite mismatches, we allow k (>0) mismatches when mapping a bisulfite read onto a reference. A genomic location is deemed to be unique for a read if it is the only location with the lowest number of mismatches with respect to the read.

### Evaluation on the simulated Illumina data

**Figure 2:**
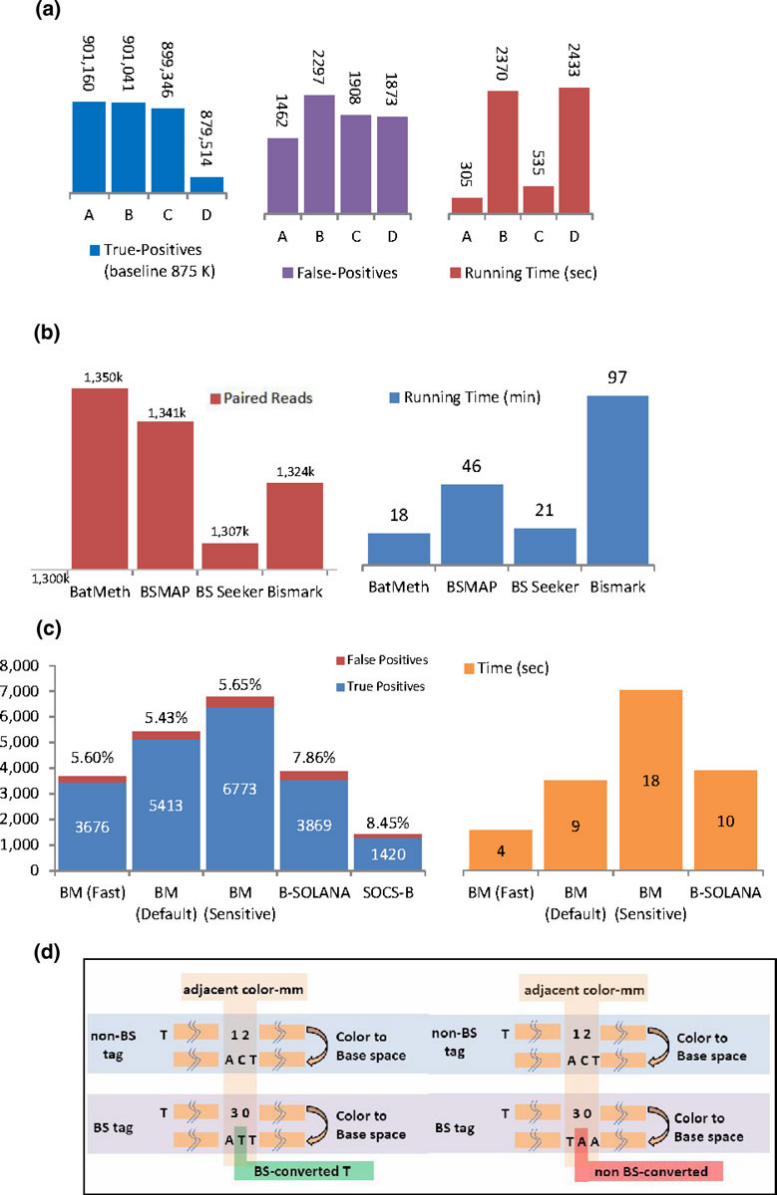
**Benchmarking of programs on various simulated and real data sets**. **(a) **Benchmark results of BatMeth and other methods on the simulated reads: A, BatMeth; B, BSMAP; C, BS-Seeker; D, Bismark. The timings do not include index/table building time for BatMeth, BS-Seeker, and Bismark. These three programs only involve a one-time index-building procedure but BSMAP rebuilds its seed-table upon every start of a mapping procedure. **(b) **Insert lengths of uniquely mapped paired reads and the running times for the compared programs. **(c) **Benchmark results on simulated SOLiD reads. Values above the bars are the percentage of false positives in the result sets. The numbers inside the bars are the number of hits returned by the respective mappers. The graph on the right shows the running time. SOCS-B took approximately 16,500 seconds and is not included in this figure. **(d) **bisulfite and non-bisulfite induced (SNP) adjacent color mismatches.

We generated 1 million reads, each 75 bp long, which were randomly simulated from the human genome hg19 using the simulator found in RMAP-bs [31]. The data set was built by allowing a maximum of three mismatches per read. Each C in the simulated read, regardless of its context, was bisulfite converted at a uniform rate of 97%. We benchmarked BatMeth and the other methods, BSMAP, BS-Seeker and Bismark, on this data set (see Section 1.1 of Additional file 1 for parameters used). Since the original coordinates in the simulated reads are known, we can evaluate the accuracy of all the programs by comparing their outputs with the original coordinates. We mapped the reads onto the reference allowing at most three mismatches. BatMeth recovered the most number of true positives and the lowest number of false positives and is the fastest program, as shown in Figure 2a.

We further illustrate that BatMeth can achieve better unbiased methylation calls than the best published method, Bismark, by replicating the experimental settings of Figure 2b in [27]. We used the same simulator, Sherman [32], the same number of reads (1 million), the same length of read (75 bases) and the same reference genome (NCBI37) for this comparison. We used Sherman to simulate 11 sets of data, from 0% to 100% of bisulfite conversion in increments of 10%. Sherman emulates bisulfite conversion by converting all Cs regardless of their genomic context with a uniform distribution. No non-bisulfite mismatches were allowed in the reads, during the scanning phase, for both BatMeth and Bismark. The results produced by Bismark show exactly the same trends as the graph that was presented in [27]. Table 1 presents the performance of BatMeth and Bismark in terms of mapping efficiency, detected methylation levels in different genomic contexts from various *in silico *methylation rates in different contexts (CG, CHG and CHH genomic contexts, where H stands for base A/C/T only). BatMeth has an average of approximately 1.1% better mapping efficiency and about twice the accuracy as Bismark in estimating methylation levels of Cs from different genomic contexts with different initial methylation levels.

**Table 1:** Comparison of mapping efficiencies and estimation of methylation levels in various genomic contexts

BatMeth (%)				Bismark (%)				
		
Mapping efficiency	CG	CHG	CHH	Mapping efficiency	CG	CHG	CHH	Oracle BS rate (%)
94.2	0.0	0.0	0.0	91.1	0.0	0.0	0.0	0.0
94.0	10.0	10.0	10.0	92.1	10.0	10.0	10.0	10.0
93.9	20.0	20.0	20.0	92.4	20.0	20.1	20.0	20.0
93.8	30.0	30.0	30.0	92.5	29.9	30.0	30.0	30.0
93.6	39.9	40.0	40.0	92.5	40.0	40.0	40.0	40.0
93.5	50.0	50.0	50.0	92.6	50.0	50.0	50.0	50.0
93.4	60.0	60.0	60.0	92.6	60.0	60.1	60.0	60.0
93.2	70.0	70.0	70.0	92.7	70.0	70.0	70.0	70.0
93.0	79.9	80.0	80.0	92.6	79.9	80.0	80.0	80.0
92.8	90.0	90.0	90.0	92.6	90.1	90.0	90.0	90.0
92.6	100	100.0	100.0	92.6	100.0	100.0	100.0	100.0

Methylation levels in various genomic contexts, such as CG, CHG and CHH (H is A/C/T only), are called by BatMeth and Bismark and validated against the oracle bisulfite rate used in Sherman.

### Evaluation on the real illumina data

We downloaded about 850 million reads sequenced by Illumina Genome Analyzer II (Gene Expression Omnibus (GEO) accession number [GSE19418]) [33] on H9 embryonic stem cells. Since BSMAP is not efficient enough to handle the full data set, 2 million paired-end reads were randomly extracted from one of the runs in [GSE19418] for comparative analysis with BSMAP. Reads were observed to have a lot of Ns near the 3' end and were trimmed down to 51 bp before being mapped onto hg19 with at most two mismatches per read (see Section 1.2 of Additional file 1 for parameters used).

For this sample data set, BatMeth mapped 1,518,591 (75.93%) reads uniquely compared to 1,511,385 (75.57%) by BSMAP, 1,474,880 (73.74%) by BS-Seeker and 1,498,451 (74.92%) by Bismark. Out of all the hits reported by BatMeth, 1,505,190, 1,464,417 and 1,481,251 mapped loci were also reported by BSMAP, BS-Seeker and Bismark, respectively. BatMeth found 13,401, 54,174 and 37,340 extra hits when compared to BSMAP, BS-Seeker and Bismark, respectively. BSMAP, BS-Seeker and Bismark also found 6,195, 10,463 and 17,220 extra hits, respectively, when compared to our result set.

Next, we mapped the two reads of every paired-end read independently to investigate the mapping accuracy of the compared programs. Since the insert size of this set of paired-end reads is approximately 300 bp, a pair of partner reads can be expected to be mapped correctly with a high probability if they are mapped concordantly within a nominal distance of 1,000 bp. The high number of such pairable reads (Figure 2b) indicates that BatMeth is accurate. Figure 2b also shows that BatMeth is fast.

We have also downloaded approximately 28.5 million reads sequenced by Illumina Genome Analyzer II on the human H1 embryonic cell line (GEO accession numbers [SRR019048], [SRR019501] and [SRR019597]) [20]. We only compared BatMeth with BS-Seeker since BSMAP and Bismark are too slow (see Section 1.3 of Additional file 1 on parameters used). Furthermore, Krueger and Andrews [21] mention that Bismark is both slower and less likely to report unique hits than BS-Seeker. Table 2 shows the unique mapping rates and running times of BatMeth and BS-Seeker. In summary, BatMeth achieved the best mappability rate, lowest estimated false positive rate and was the fastest on real Illumina data.

**Table 2:** Comparison of speed and unique mapping rates on three lanes of human bisulfite data

	Number of reads	Unique mapping (%) ^a^		Running time (minutes) ^a^	
			
Read file		BatMeth	BS-Seeker	BatMeth	BS-Seeker
SRR019048	15,331,851	37.4	37.2	30	87
SRR019501	7,217,883	44.7	44.5	16	41
SRR019597	5,943,586	58.2	58.1	13	37

^a^Threshold of two mismatches used.

### Evaluation on the simulated SOLiD data

We generated 10,000 simulated reads, each having 51 color bases, that were randomly extracted from chromosome 1 of UCSC hg19 using the simulator from RMAP-bs [31]. RMAP-bs was used to convert the Cs in the reads, regardless of its context, to Ts at a uniform rate of 97% to simulate bisulfite conversions. In addition, for each read, zero to two non-bisulfite base mismatches were introduced with equal chance before the read was converted to color space. Lastly, sequencing errors were added at a uniform rate of 5% to the reads.

The simulated color reads were mapped using BatMeth, SOCS-B and B-SOLANA allowing resultant unique hits to have at most three mismatches. Precisely, BatMeth and SOCS-B allowed at most three non-bisulfite mismatches while B-SOLANA did not discount bisulfite mismatches (see Section 1.4 of Additional file 1 for parameters used). Figure 2c summarizes the results of the three programs together with the verification against the oracle set. BatMeth gave many more correct hits and fewer wrong hits than both SOCS-B and B-SOLANA. BatMeth can be made to offer a flexible tradeoff between unique mapping rates and speed. In the 'default' mode, BatMeth was found to be more sensitive (approximately 15%) and faster (approximately 10%) than the most recent published B-SOLANA. In the 'sensitive' mode, BatMeth was found to be more sensitive (approximately 29%) and slower (approximately two times) than B-SOLANA. In addition to producing approximately 15% to 29% more correct hits, BatMeth had a precision of 94.5% while that of B-SOLANA and SOCS-B was 92.1% and 91.5%, respectively. These statistics show that BatMeth is an accurate mapper for color reads.

**Figure 3:**
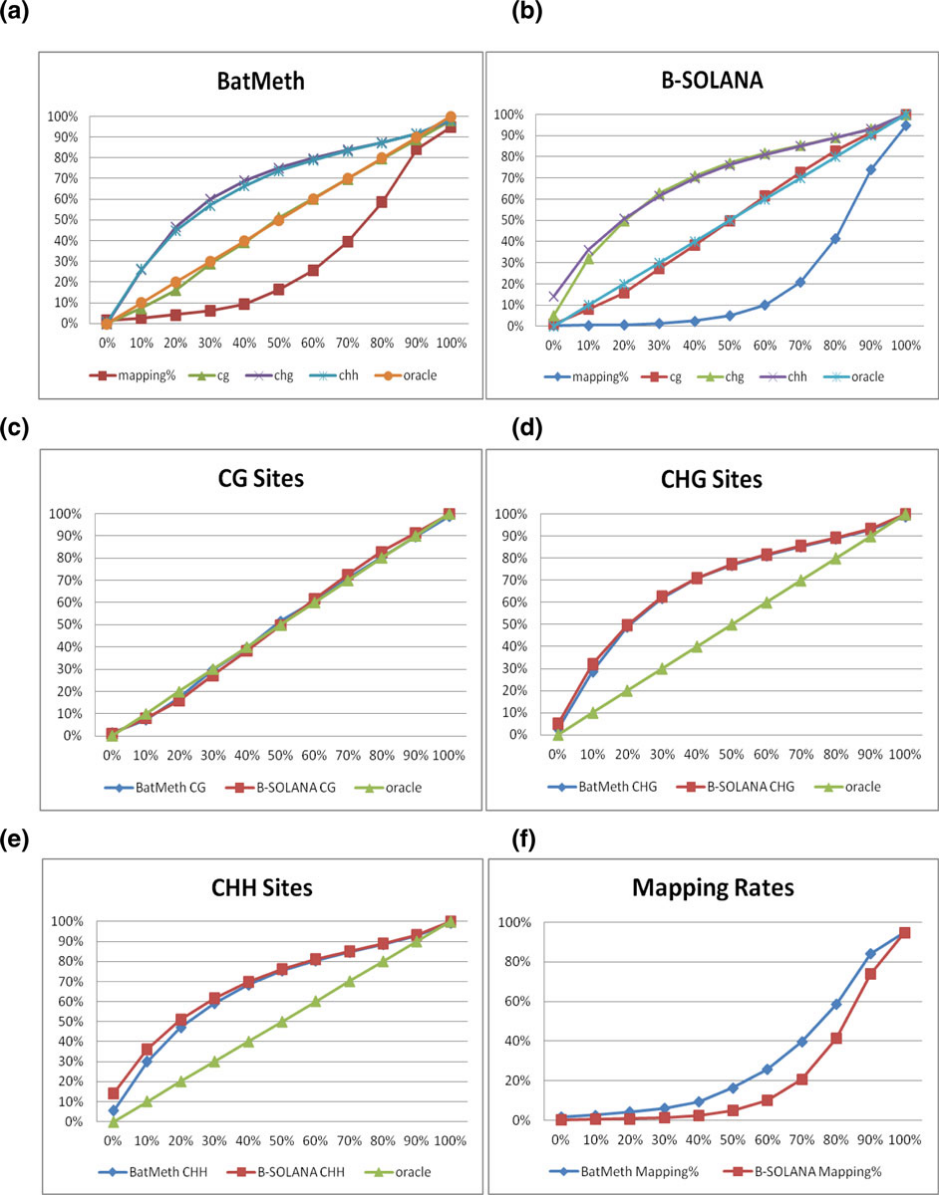
**A total of 10^6^, 75 bp long reads were simulated from human (NCBI37) genomes**. Eleven data sets with different rates of bisulfite conversion, 0% to 100% at increments of 10% (context is indicated), were created and aligned to the NCBI37 genome. **(a-e) **The x-axis represents the detected methylation conversion percentage. The y-axis represents the simulated methylation conversion percentage. **(f) **The x-axis represents the mapping efficiency of the programs. The y-axis represents the simulated methylation conversion percentage of the data set that the program is mapping. (a,b) The mapping statistics for various genomic contexts and mapping efficiency with data sets at different rates of bisulfite conversion for BatMeth and B-SOLANA, respectively. (c-e) Comparison of the methylated levels detected by BatMeth and B-SOLANA in the context of genomic CG, CHG and CHH, respectively. **(f) **Comparison of mapping efficiencies of BatMeth and B-SOLANA across data sets with the described various methylation levels.

To illustrate that BatMeth can achieve better unbiased methylation calls for color reads than the best published method, B-SOLANA, we replicated the experimental settings of Figure 2c in [27] to compare the two programs; we used the same simulator (Sherman), the same number of reads (1 million), the same length of read (75 bp) and the same reference genome (NCBI37) for this comparison. We used Sherman to simulate 11 sets of data, from 0% to 100% of bisulfite conversion at increments of 10%. Sherman emulates bisulfite conversion by converting all Cs regardless of their genomic context with a uniform distribution. Default parameters were used for BatMeth and B-SOLANA. The graph produced by us for B-SOLANA shows the same trends as that presented in [27]. We further broke down the graphs as well as those in Figures 3a (BatMeth) and 3b (B-SOLANA), which show rates of methylation calling for various *in silico *methylation rates (0% to 100% at divisions of 10% of bisulfite conversion) in different contexts (CG, CHG and CHH genomic contexts, where H stands for base A/C/T only) of the genomes, into separate series of data. Subsequently, we did a direct comparison between BatMeth and B-SOLANA to show that BatMeth is better than B-SOLANA in all contexts of methylation calling, namely, CG (Figure 3c), CHG (Figure 3d), CHH (Figure 3e) and non-unique mapping rates (Figure 3f). To be exact, BatMeth was approximately 0.7%, 0.7% and 2.2% more accurate than B-SOLANA in the methylation callings of the CG, CHG and CHH sites, respectively, and had an average of approximately 9.2% more non-unique mappings than B-SOLANA on the tested data sets.

### Evaluation on the real SOLiD data

We downloaded about 495 million reads sequenced by AB SOLiD system 3.0 (Sequence Read Archive (SRA) accession number [SRX062398]) [13] on colorectal cancer. Since SOCS-B is not efficient enough to handle the full data set, 100,000 reads were randomly extracted from [SRR204026] to evaluate BatMeth against SOCS-B and B-SOLANA. The mismatch threshold used was 3 (see Section 1.5 of Additional file 1 for parameters used).

Table 3 compares the unique mapping rates and running times between BatMeth, SOCS-B and B-SOLANA. Note that BatMeth always has a higher unique mapping rate (from 39.6% to 52.1%; from fast to sensitive mode) than the next best method, B-SOLANA with 37.4%. At the same time, BatMeth maintained low rates of noise (from 0.47% to 1.75%; from fast to sensitive mode). Hence, it is still more specific than the other programs. In terms of running time, BatMeth fast mode is approximately 1.7 times faster and BatMeth sensitive mode is approximately 4 times slower than B-SOLANA. It was also observed that 3.26% of the resultant hits from B-SOLANA are duplicated; some of the reads were given two hit locations as B-SOLANA traded speed for checking the uniqueness of hits.

**Table 3:** Unique mapping rates and speed on 100,000 real color reads

SRR204026	Unique mapping (%)^a^	Estimated noise (%)^b^	Timing
BatMeth (fast)	39.6	0.47	77 s
BatMeth (default)	45.8	0.94	247 s
BatMeth (sensitive)	52.1	1.75	521 s
B-SOLANA^c^	37.4	2.06	130 s
SOCS-B^d^	28.3	4.55	~71 h

^a^We tabulated the unique mapping rates of the 100,000 reads. ^b^The error rates are estimated from the number of reverse-strand mappings as stated by Equation 2 in Materials and methods. ^c^Note that 3.26% of B-SOLANA's resultant reads are double-counted as B-SOLANA reported two hits for them. One of the two hits is assumed to be correct for the estimation of the noise rate of B-SOLANA. ^d^Reverse-strand mapping is allowed by enabling G-A transitions in SOCS-B. BatMeth fast, default, and sensitive modes were run with -n0-N3, -n0-N4, -n0-N5 as parameters, respectively.

Based on the experiments performed, BatMeth's memory usage peaked at 9.3 GB (approximately 17 seconds of load time) for Illumina reads and 18.8 GB (approximately 35 seconds of load time) for color reads while BSMAP and BS-Seeker peaked at 9+ GB and Bismark peaked at 12 GB. SOCS-B peaked at 7+ GB and B-SOLANA peaked at 12 GB. Parameters used for all experiments are recorded in Additional file 1. In summary, the experiments in this section show that BatMeth is the fastest among all the compared programs. Furthermore, BatMeth also has the highest recovery rate of unique hits (exclusive of false positives) and the best accuracy among all the compared programs.

## Discussion

DNA methylation is an important biological process. Mapping the bisulfite reads from next-generation sequencing has enabled us to study DNA methylation at single-base resolution. This paper aims to develop efficient and accurate methods to map bisulfite reads.

This study employed three methods to evaluate the performance of bisulfite read mapping methods. The first method measured the ratio of correct and wrong unique unambiguous mappings. This method only applies to simulated data when the actual locations of the reads are known. For real data, the number of unambiguous mappings alone may not be a good criterion to evaluate accuracy (we can map more reads at a higher mismatch number, which results in lower specificity). The second method evaluated the accuracy using the number of reads that were mapped in consistent pairs, and can only be employed when paired-end read information is available. The third method used the directionality of the mapped reads from SOLiD sequencing. For the SOLiD reads, we mapped reads unbiasedly onto both forward and reverse directions of our reference genome. From the unambiguous mappings, we estimated the error rate of our unique mappings from the proportion of reverse direction unique mappings in the result sets. All these measures were used on different sets of simulated and real data and they suggest that BatMeth produces high quality mapping results.

For future work, our team will be working on more time-efficient data structures to better streamline our algorithm.

## Conclusions

We report a novel, efficient and accurate general-purpose bisulfite sequence mapping program. BatMeth can be deployed for the analysis of genome-wide bisulfite sequencing using either base reads or color reads. It allows asymmetric bisulfite conversion to be detected by labeling the corresponding reference genome with the hit. The components discussed in the Materials and methods section, such as *List Filtering*, *Mismatch Stage Filtering*, *Fast Mapping onto Two Indexes*, *Handling Hypo- and Hyper-Methylation Sites *and *other heuristics *have offered increased speed and mappability of reads. In addition, BatMeth reduces biased detection of multiple CpG heterogeneous and CpH methylation across the whole reference by mapping onto both fully converted and non-CpG references and then labeling the reference to which the hits are from to aid biologists to discriminate each hit easily. Users can also choose to bias against either reference with varying mismatch scans. In assessing the uniqueness of a hit for bisulfite color reads, BatMeth considers both strands of the DNA simultaneously while B-SOLANA considers both DNA strands separately. Hence, BatMeth has a stronger uniqueness criterion for hits as B-SOLANA may produce two hits for a read, one hit for each separate DNA strand. Lastly, BatMeth uses an optimal dynamic programming algorithm to convert the color read to base space to check for non-bisulfite mismatches.

## Materials and methods

### Methods for base reads

#### Problem definition and overview of the method

The problem of mapping bisulfite reads is defined as follows. A bisulfite treatment mismatch is defined as a mismatch where the aligned position is a T in the read and the corresponding position in the reference genome is a C. Given a set of bisulfite reads, our task is to map each bisulfite read onto the reference genome location, which minimizes the number of non-bisulfite mismatches.

**Figure 4:**
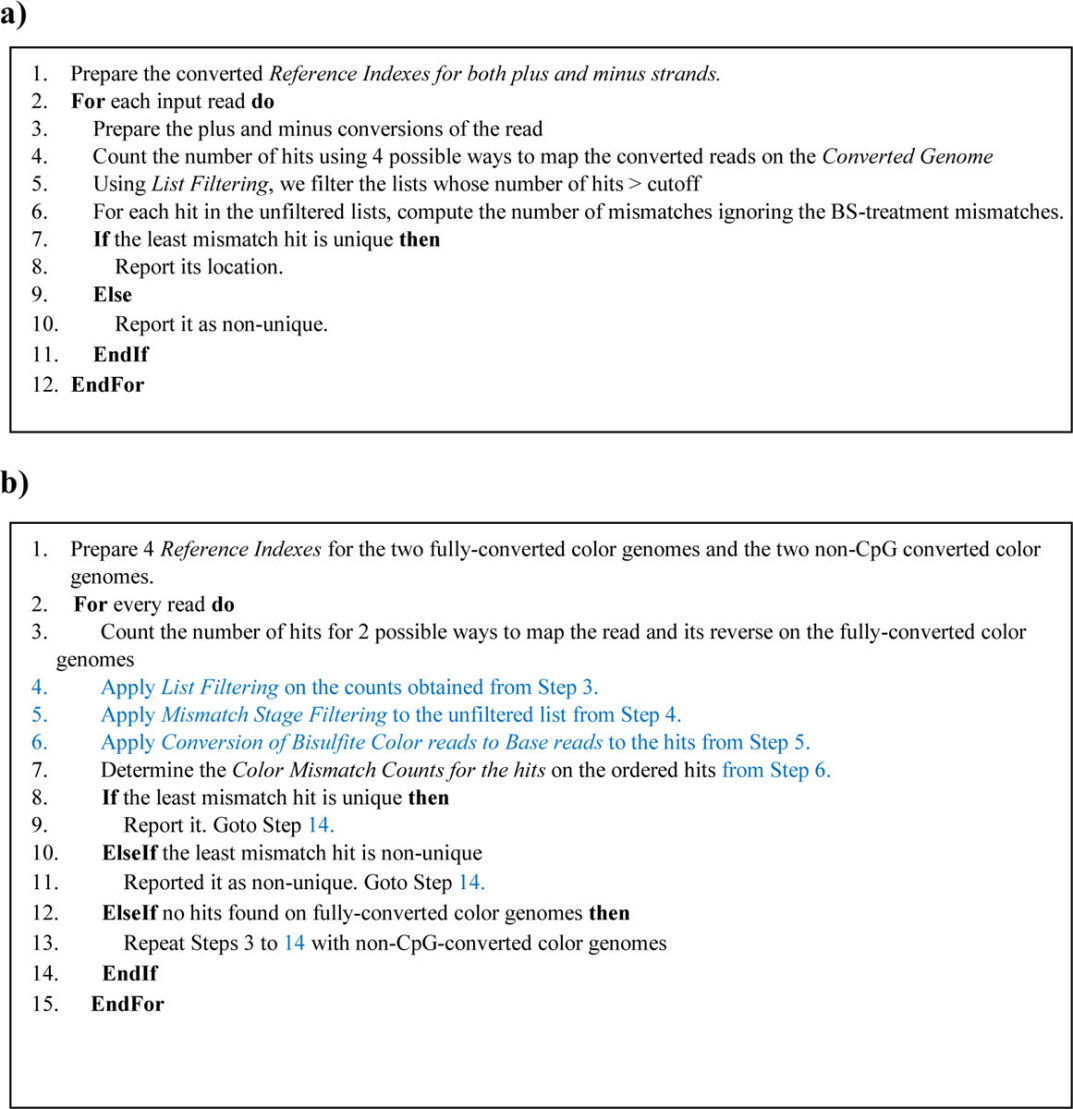
**Outline of the mapping procedure**. **(a) **Mapping procedure on Illumina bisulfite base reads. **(b) **Mapping procedure on SOLiD color-space bisulfite reads.

The algorithm of BatMeth is as follows. BatMeth starts off by preparing the *Converted Genome *and does a one-time indexing on it. Next, *Low Complexity BS reads *will be discarded; otherwise, we will do a *Counting Hits of BS Read *on them and discard the hits according to *List Filtering*. After this, each of the retained hits will be checked for bisulfite mismatches by ignoring C to T conversions caused by the bisulfite treatment. BatMeth reports the unique hit with the lowest non-bisulfite mismatches for each read. Figure 4a outlines the algorithm and we discuss the novel components that aid BatMeth to gain speed and accuracy below.

#### Converted Genome

Similar to BS-Seeker and Bismark, we prepare a converted reference genome with all Cs converted to Ts. Since the plus and minus strands are not complementary after Cs are converted to Ts, we have to create two converted references where one is for the plus strand and the other is for the minus strand. Burrows-Wheeler transform (BWT) indexing of the two new converted references is done before the mapping.

#### Low Complexity BS reads

BatMeth does not map bisulfite reads with low complexity. The complexity of the raw read is computed as Shannon's entropy, and raw bisulfite reads with a differential entropy *H *< 0.25 are discarded. In BatMeth, differential entropy is estimated from the discrete entropy of the histogram of A/C/G/T in a read. Depending on the design of the wet-lab experiment, the amount of reads being discarded by this entropy cutoff varies. In our experiments on Illumina reads, approximately 0.5% of the reads were discarded.

#### Counting Hits of BS read and List Filtering

For those reads that pass the complexity filter, we first convert all Cs to Ts and map them against the converted genomes. In contrast to existing methods, BatMeth does not obtain the best or second best hits (for example, BS-Seeker and Bismark) from each possible orientation of a converted read and reports the lowest-mismatch locus to be the resultant hit for a read. In the case of hyper-methylation, the correct hit may not be the best or second best hit as it might contain more mismatches. Thus, this approach will miss some correct solutions. BatMeth also does not enumerate all hits like BSMAP, which is slow. Instead of mapping the reads directly, BatMeth counts the number of hits where the read or its reverse complement can occur on the two converted genomes using an in-house short read mapper, BatMis Aligner [34]. Table 4 shows the four ways of aligning the converted reads onto the converted genomes, which yield four counts of hits.

**Table 4:** Possible ways to map a bisulfite read onto the converted genome

	Reference (C→T)	RC reference (C→T)
Read (C→T)	Count 1	Count 2
RC Read (C→T)	Count 3	Count 4

RC, reverse-complement.

Out of the four counts on the four lists, only one list contains the true hit. List filtering aims to filter away those spurious lists of hits (represented by the counts) that are unlikely to contain the true hit. Note that a read can appear to be repetitive on one strand but unique on the opposite strand of the DNA. Hence, if a list has many hits (by default the cutoff is set to be 40 hits) with the same number of mismatches, we discard such a list since it is likely to be spuriously reported for one strand of the reference genome. Another reason for rejecting such lists is that they may contain hits that may be of the same mismatch number as the hit that is unique on the opposite strand, rendering all hits as ambiguous.

Apart from improving the uniqueness of the putative resultant hit among all reported hits of a bisulfite read, filtering also reduces the number of candidate hits that need to be checked. This improves the efficiency of the algorithm. For example, consider the simulated bisulfite-converted read 'ATATATATGTGTATATATATATATATATATATGTGTATATATATGTGTGTATATATATATA TATATATGTATATAT' being mapped onto the converted hg19 genomes as discussed earlier. We obtained four counts of 1, 0, 40 and 40 hits by mapping the converted reads onto the converted genomes. The last two lists are filtered away since they have too many hits, leaving us to check only one hit instead of 81 for bisulfite mismatches. Since the data are simulated, the unfiltered hit is found to be the correct unique hit for this read, which the other mappers cannot find.

Table 5 shows the effect of using *List Filtering *on the same set of simulated data from *Evaluation on the Simulated Illumina Reads*. We ran BatMeth with different cutoffs for *List Filtering *and we can see that the time taken increased linearly with increasing cutoffs for *List Filtering *while sensitivity and accuracy dropped. With large cutoffs such as ≥500 (marked by asterisks in Table 5), the number of wrong hits increased while sensitivity still continued to drop. Thus, we have chosen a cutoff of 40 for a balance of speed, sensitivity and accuracy. (Disabling *List Filtering *will cause BatMeth to check through all the reported candidate locations for a read and will slow BatMeth down by approximately 20-fol fold, as shown in Table 5.)

**Table 5:** Cutoffs for list filtering on simulated reads from the Results section

List size	Mismatch counting in seconds^a^	Correct hit	Wrong hit	Total hit
20	136	901,164	1,516	902,680
40	165	901,160	1,462	902,622
60	191	901,165	1,454	902,619
100	279	901,166	1,448	902,614
200	475	901,166	1,447	902,613
500	1,197	901,167	1,450*	902,617
1,000	2,942	901,167	1,450*	902,617

Asterisks indicate increased false-positives produced with large list filtering cutoffs.

### Methods for color reads

#### Overview of the method

Due to the di-nucleotide encoding and sequencing errors in SOLiD color reads, a naïve conversion from color space to base space is hardly possible without errors. As a color error in a read will introduce cascading base-space errors, we cannot use the method described in *Methods for Base Reads *to map bisulfite color reads. This section describes how we aim to map each bisulfite color read uniquely to the reference genome while minimizing the number of non-bisulfite treatment mismatches.

The algorithm of BatMeth is as follows. BatMeth starts by preparing *Converted Genome *and *Non-CpG Converted Genome*, and does a one-time BWT indexing on them. For every color read, we do a *Counting Hits of BS Color Read *of the read on the references and discard them according to *List Filtering*. After applying *Mismatch stage Filtering*, the unfiltered hits are converted to base space as described in *Conversion of Bisulfite Color Reads to Base Reads *to allow for the checking of bisulfite-mismatches. The *Color Mismatch Count *for the retained hits is then determined and the unique locus with the lowest mismatch count reported; otherwise, no hits are reported for this read. We have also utilized additional heuristics, such as *Fast Mapping onto Two Indexes *and *Handling Hypo- and/or Hyper Methylation Sites *to speed up and improve the accuracy of BatMeth, which we discuss below. All the components, namely, *List Filtering*, *Mismatch Stage Filtering, Conversion of Bisulfite Color Reads to Base Reads, Color Mismatch Count, Fast Mapping onto Two Indexes and Handling Hypo- and/or Hyper Methylation Sites *differ from existing methods. Figure 4b outlines the algorithm and shows how the components are assembled for SOLiD color-space bisulfite read mapping.

#### Non-CpG Converted Genome

The reference genome and its reverse-complement were first prepared by converting all its Cs to Ts as described in the base reads mapping procedures; then, the two converted genomes are encoded into color space. These two genomes are called fully converted color genomes. In addition, the reference genome and its reverse-complement are similarly converted except that the Cs in CpG are left unchanged. We call these the non-CpG converted color genomes. Finally, the BWT indexes for these four color genomes are generated.

In the algorithm, the bisulfite color reads will be mapped to the fully converted color genomes to identify unique hits first; if this fails, we will try to map the reads onto the non-CpG converted color genomes and BatMeth will label which reference a hit is from.

The reason for using the non-CpG converted genome is that the conversion step for bisulfite color reads is different from that for Illumina. In Illumina reads, the C-to-T mismatches between the raw bisulfite reads and the reference genome are eliminated by converting all Cs to Ts in both the reads and the reference genomes. However, we cannot make such a conversion in bisulfite color reads as we do not know the actual nucleotides in the reads. Based on biological knowledge, we know that CpG sites are expected to be more methylated [35]. Hence, such conversion reduces the number of mismatches when the color reads are mapped onto the reference genome in color space. This aids in gaining coverage in regions with high CpG content. Thus, BatMeth maps bisulfite reads to both hyper- and hypo-methylation sites.

#### Counting Hits of BS-Color Read and List Filtering

Unlike sequencing by Illumina, SOLiD only sequences reads from the original bisulfite-treated DNA strands. During PCR amplification, both strands of the DNA are amplified but only the original forward strands are sequenced. Subsequently, during the sequencing phase, reverse-complement reads are non-existent as a specific 5' ligated P1 adaptor is used. As such, matches to the reverse-complement of the bisulfite-converted reference genome are invalid.

In other words, although a bisulfite color read has four possible orientations to map on the non-CpG converted color genomes (or the fully converted color genomes), only two orientations are valid as opposed to the four orientations in the pipeline on Illumina reads (Table 6). As opposed to the mapping of Illumina reads, it is not preferred to do a naïve conversion of color reads to base space prior to mapping. Figure 1a shows that a single base call error in an Illumina read will introduce one mismatch with respect to the reference. However, Figure 1b shows that a single base color call error in a color read will introduce cascading base mismatches instead of just one color mismatch if we are to map the color read as it is onto the reference in color space.

**Table 6:** Possible ways to map a bisulfite color read onto the converted color genome

	Reference (C→T)	RC reference (C→T)
Read	Count 1	Invalid
RC read	Invalid	Count 4

RC, reverse-complement.

Thus, we will need to do a primary map onto a converted genome with a higher mismatch parameter (by default, 4) than what we usually use for Illumina bisulfite reads as a bisulfite mismatch will introduce two adjacent color mismatches (see Figure 1c for an example of bisulfite-induced adjacent color mismatches). Similar to mapping Illumina reads, we count the number of possible hits from the two valid orientations. Then, the *List Filtering *step is applied to filter the lists with too many hits (by default, more than 10). (Note that this property also helps us to estimate the noise rate; we discuss this further in *Noise Estimation in Color-reads*.

#### Conversion of Bisulfite Color Reads to Base Reads

After the color bisulfite reads are aligned to the reference genome, we can convert the color bisulfite reads to their most-likely nucleotide equivalent representation. In the context of bisulfite mapping, we discount all the mismatches caused by bisulfite conversions.

We use a dynamic programming formulation as presented in [36] to convert color reads to base reads except that the costs for bisulfite-induced mismatches have to be zeroed when the reference is C and the read is T. This conversion is optimal and we use the converted base read to check against the putative genomic locations from *List Filtering *to interrogate all mismatches in the read to determine if they are caused by bisulfite conversion, base call error or SNP.

#### Color Mismatch Count

After converting each color read to its base-space equivalent representation, we can calculate the number of base mismatches that are actually caused by bisulfite treatment in the color read. Figure 2d shows two different types of adjacent color mismatches that are caused by bisulfite conversion (left) and non-bisulfite conversion (right). For bisulfite-induced adjacent mismatches, we assign a mismatch cost of 0 to the hit. For non-bisulfite-induced adjacent mismatches, we assign a mismatch cost of 1 to the hit.

To be precise, we consider a color read as C[1..L], where L is the read length, and let B[1..L-1] be the converted base read computed from the dynamic programming described previously and mm[i] as a mismatch at position i of C, which is computed using Equation 1. The mismatch count of C is computed as mm[1]+...+mm[L-1], where:

(1) $$mm\left[i\right]=\left\{\begin{array}{cc} 1, & if\;C\left[i\right]\;and\;C\left[i+1\right]\text{ are color mismatches},\;B\left[i\right]\text{ is non-BS mismatch}\\ 0, & otherwise \end{array}\right)$$

#### Mismatch Stage Filtering

We have developed a set of heuristics to improve the rate of finding a unique hit among the set of candidate hits. First, we sort and group the initial hits by their number of color mismatches; then, we try to find a unique hit with the minimum non-bisulfite-mismatch count within each group of hits.

As the bound of color mismatches is known, we can apply a linear time bucket sort to order all the candidate hits according to their mismatch counts. The group of initial mapping loci with the lowest mismatch number is recounted for their number of base mismatches using the converted read in base space obtained from the previously discussed dynamic programming formulation. If a unique lowest base mismatch hit exists among them, we report this location as unique for this read. Otherwise, we proceed to recount the base mismatches for the group of mapping loci with the next highest color mismatch count. We continue this procedure until a unique hit is found or until there are no more color-space mismatch groups to be examined. A unique hit must be unique and also minimizes the base mismatch counts among all previously checked hits in the previous groups.

Mismatch stage filtering enables us to check less candidate hits, which speeds up the algorithm. It also improves the unique mapping rate as there are less ambiguous hits within a smaller group of candidate hits.

When the above components are applied, the mapping rates on SOLiD data improve progressively as seen below. By using Equation 1 to count color mismatches, BatMeth was able to increase the number of unique mappings by approximately 9% and by employing *Mismatch Stage Filtering*, unique mapping rate is approximately increased by another 3%. With this increase in unique mappings of approximately 12%, BatMeth had an estimated noise level of approximately 1% as based on Equation 2 while B-SOLANA and SOCS-B had an estimated noise levels of approximately 2.06% and 4.55%, respectively, on the same set of 100,000 reads. These statistics agree with the results on the simulated data and indicate that BatMeth is capable of producing low-noise results.

#### Fast Mapping onto Two Indexes

As mentioned in *Non-CpG Converted Genome*, we map bisulfite color reads onto four converted references, two of which have their Cs converted to Ts at non-CpG sites and the other two have all their Cs converted to Ts. It was observed that mappings on both non-CpG converted and fully converted references highly coincide with each other with an approximately 95.2% overlap. Due to this observation, we try to map onto the fully converted reference first to give us a mapping to regions of hypo-methylation status. If there are no mappings found on the fully converted references, then BatMeth maps the same read again onto the non-CpG converted references, which biases hyper-methylation sites. This allows the simultaneous interrogation of canonical CpG hyper-methylation sites with reduced biased mapping on the fully converted genome. BatMeth also labels each hit with the type of converted references it was mapped to. Overall, this approach can save time by skipping some scanning of the non-CpG-converted references.

#### Handling Hypo- and/or Hyper-Methylation Sites

With prior knowledge of the methylation characteristics of the organism to be analyzed, different *in silico *conversions to the reference can be done and the best alignments can be determined from the combined set of results of different mapping runs. BatMeth uses two types of converted genomes to reduce mapping biases to both hyper- and hypo-methylation sets. Since the two sets of hits from the two genomes coincide to a large extent, we can save time by scanning a read on one genome with a much lower mismatch number than on the other genome.

BatMeth allows users to choose the mismatch number they want to scan on each of the two types of genomes. We now introduce M1 and M2 (capped at 5) as the mismatch numbers used in the scans against the fully converted and non-CpG-converted genomes, respectively. For the best sensitivity, BatMeth scans at M1 = M2 = 5 for both hyper- and hypo-methylation sites. For the highest speed, BatMeth scans at [M1 = 0, M2 = 3] and [M1 = 3, M2 = 0], which will perform biased mapping to hyper- and hypo-methylation at CpG sites, respectively. Figure 2c shows the results of running the various modes of BatMeth (Fast, Default and Sensitive) on a set of 10,000 simulated color reads.

#### Noise Estimation in Color-reads

To estimate noise rates, we map the real reads in their two possible orientations onto the genome. If a hit is found for a read from the original strands of the genome, we try to map the same read onto the complement strand of the genome too. If a lower mismatch hit can be found from the complement strand of the genome, then we mark the result for this read as noise. We use the proportion of marked reverse-complement unique mappings to estimate the noise level, given by Equation 2:

(2) $$err=\frac{\#\;of\;reverse\text{-}complement\;mappings}{\#\;of\;mappings}$$

#### Handling Ambiguous Bases

For base reads, non-A/C/G/T bases are replaced by A so they will not affect the callings of methylation sites. Similarly, color reads with non-A/C/G/T bases are replaced with 0. Non-A/C/G/T bases on the reference genome are converted to A to avoid affecting downstream methylation callers. We have avoided converting them to random nucleotides as it may produce false hits in regions containing ambiguous bases. We mapped 1 million 75 bp reads and have seen reads being mapped to poly-N regions. This can be mostly attributed to the reduced alphabet size, from four to three, due to bisulfite conversions.

## Abbreviations

bp: base pair; BS: bisulfite; BWT: Burrows-Wheeler transform; C: cytosine; GB: gigabyte; GEO: Gene Expression Omnibus; SNP: single-nucleotide polymorphism; T: thymine.

## Competing interests

The authors declare that they have no competing interests.

## Authors' contributions

SWK oversaw the project. LJQ and SWK designed the algorithm and wrote the paper. SWK, LJQ and LGL designed the experiments and revised the paper. LJQ and CT implemented BatMeth. WCL, RYJ and EW designed the wet-lab experiments for benchmarking. All authors have read and approved the manuscript for publication.

## Supplementary Material

Additional file 1 **Chosen parameters**. This file details the parameters used by the various programs in the Results section. Click here for file
