# Supplementary material for: BatMeth: improved mapper for bisulfite sequencing reads on DNA methylation
Source: Genome Biol. 2012 Oct 3;13(10):R82. doi: 10.1186/gb-2012-13-10-r82 (PMC3491410; doi:10.1186/gb-2012-13-10-r82)
Supplement: Additional file 1 — Chosen parameters. This file details the parameters used by the various programs in the Results section. [file gb-2012-13-10-r82-S1.docx]

Supplementary Notes

BatMeth: Improved Mapper for Bisulfite Sequencing Reads on DNA Methylation

# The Experiments

The input files can be downloaded from http://www.comp.nus.edu.sg/~limjingq/inputs.zip .

## Parameters used for Simulated Solexa Reads

For the experiments on simulated Solexa reads, we have used the input file - met_sample_rmap_simError.fa. The following parameters are used for the compared programs.

BatMeth: ./batmeth –g hg19.fa -i INPUT -n 3 -o TEMP -O 1 -p 4

./split OUTPUT hg19.fa 3 y TEMP.0 TEMP.1 TEMP.2 TEMP.3

BSMAP: ./bsmap -a INPUT -d hg19.fa -o OUTPUT -v 3 -p 4 -n 1 -f 2

Bismark: ./bismark -f -n 3 --path_to_bowtie bowtie_0.12.7/ --direction hg19/ INPUT

BS Seeker: python BS_Seeker.py -i INPUT -t N -e 75 -p bowtie_0.12.7/ -m 3

## Parameters used for Real Solexa Reads

For the experiments on real Solexa reads, we have used the input file - GHE002_2r68_2mil.fastq. The following parameters are used for the compared programs.

BatMeth: ./batmeth –g hg19.fa -i INPUT -n 2 -o TEMP -p 4

./split OUTPUT hg19.fa 2 y TEMP.0 TEMP.1 TEMP.2 TEMP.3

BSMAP: ./bsmap -a INPUT -d hg19.fa -o OUTPUT -v 2 -p 4 -n 1 -f 2

Bismark: ./bismark -q -n 2 --path_to_bowtie bowtie_0.12.7/ hg19/ INPUT

BS Seeker: python BS_Seeker.py -i INPUT -t Y -f W -r W -e 75 -p bowtie_0.12.7/ -m 2

## Parameters used for Real Solexa Reads – Time Benchmarks

For the speed benchmark between BatMeth and BS Seeker, we have downloaded Accession Number: SRR019048, SRR019501 and SRR019597 from public archival site. Parameters used are as follows.

BatMeth: ./batmeth -g hg19.fa -i INPUT -n 2 -o TEMP -O 1 –p 4

./split OUTPUT hg19.fa 2 y TEMP.0 TEMP.1 TEMP.2 TEMP.3

BS Seeker: python BS_Seeker.py -i INPUT -t Y -f W -r W -e 87 -p bowtie_0.12.7/ -m 2 (-e 76 is used for SRR019597)

## Parameters used for Simulated SOLiD Reads

For the experiments on simulated SOLiD reads, we have used the input file - rmap_sim2.csfasta.

BatMeth: ./batmeth -g hg19Chr1.fa -i INPUT -n 4 -N 0 -F 36 -o TEMP -p 4 (Fast: -n3, Sensitive: -n5)

./split OUTPUT hg19Chr1.fa 3 n TEMP.0 TEMP.1 TEMP.2 TEMP.3

SOCS-B: [r] – nonCpG-converted hg19_chr1 used, [c] INPUT, [s] 3, [t] 3, [i] yes, [m] 1, [T] 4, [v] bisulfite, [g] yes

B-SOLANA: ./bsolana map_watson -csfasta INPUT -qual stub -bowtie bowtie_0.12.7/bowtie -samtools samtools-0.1.18/samtools -index hg19/ -thread 4 -work sim_10k/ -name sim

./bsolana map_crick -csfasta INPUT -qual stub -bowtie bowtie_0.12.7/bowtie -samtools samtools-0.1.18/samtools -index hg19/ -thread 4 -work sim_10k/ -name sim

./bsolana bemap -mapped_watson Hansen_100k/ -mapped_crick sim_10k/ -samtools samtools-0.1.18/samtools -work sim_10k/

## Parameters used for Real SOLiD Reads

For the experiments on real SOLiD reads, we have used the input file - SRR204026_100k.csfasta.

BatMeth: ./batmeth -g hg19.fa -i INPUT -n 0 -N 4 -F 36 -o TEMP -p 4 (Fast: -N3, Sensitive: -N5)

./split OUTPUT hg19.fa 3 n TEMP.0 TEMP.1 TEMP.2 TEMP.3

SOCS-B: [r] – nonCpG-converted hg19 used, [c] INPUT, [s] 3, [t] 3, [i] yes, [m] 1, [T] 4, [v] bisulfite, [g] yes

B-SOLANA: ./bsolana map_watson -csfasta INPUT -qual stub -bowtie bowtie_0.12.7/bowtie -samtools samtools-0.1.18/samtools -index hg19/ -thread 4 -work Hansen_100k/ -name Hansen

./bsolana map_crick -csfasta INPUT -qual stub -bowtie bowtie_0.12.7/bowtie -samtools samtools-0.1.18/samtools -index hg19/ -thread 4 -work Hansen_100k/ -name Hansen

./bsolana bemap -mapped_watson Hansen_100k/ -mapped_crick Hansen_100k/ -samtools samtools-0.1.18/samtools -work Hansen_100k/

Note: If speed is of utmost importance then BatMeth can be run with –m 3 as an option to ./batmeth.
